# Early Mortality Stratification with Serum Albumin and the Sequential Organ Failure Assessment Score at Emergency Department Admission in Septic Shock Patients

**DOI:** 10.3390/life14101257

**Published:** 2024-10-02

**Authors:** Sang-Min Kim, Seung-Mok Ryoo, Tae-Gun Shin, You-Hwan Jo, Kyuseok Kim, Tae-Ho Lim, Sung-Phil Chung, Sung-Hyuk Choi, Gil-Joon Suh, Won-Young Kim

**Affiliations:** 1 Department of Emergency Medicine, University of Ulsan College of Medicine, Asan Medical Center, Seoul 05505, Republic of Korea; swdarkhorse@gmail.com (S.-M.K.); chrisryoo@naver.com (S.-M.R.); 2 Department of Emergency Medicine, Samsung Medical Centre, Sungkyunkwan University School of Medicine, Seoul 06351, Republic of Korea; taegunshin@skku.edu; 3 Department of Emergency Medicine, Seoul National University College of Medicine, Seoul National University Bundang Hospital, Seongnam 13620, Republic of Korea; drakejo@snubh.org; 4 Department of Emergency Medicine, CHA Bundang Medical Center, CHA University, Seongnam 13497, Republic of Korea; dreamkks@cha.ac.kr; 5 Department of Emergency Medicine, College of Medicine, Hanyang University, Seoul 15495, Republic of Korea; erthim@gmail.com; 6 Department of Emergency Medicine, Gangnam Severance Hospital, Yonsei University College of Medicine, Seoul 06273, Republic of Korea; emstar@naver.com; 7 Department of Emergency Medicine, College of Medicine, Korea University, Guro Hospital, Seoul 08308, Republic of Korea; kuedchoi@korea.ac.kr; 8 Department of Emergency Medicine, Seoul National University Hospital, Seoul 03080, Republic of Korea; suhgil@snu.ac.kr

**Keywords:** septic shock, SOFA score, albumin, prognosis, risk stratification

## Abstract

**Background:** Early risk stratification is crucial due to septic patients’ heterogeneity. Serum albumin level may reflect the severity of sepsis and host status. This study aimed to evaluate the prognostic ability of the initial sequential organ failure assessment (SOFA) score alone and combined with serum albumin levels for predicting 28-day mortality in patients with septic shock. **Methods:** We conducted an observational study using a prospective, multicenter registry of septic shock patients between October 2015 and May 2022 from 12 emergency departments in the Korean Shock Society and the results were validated by examining those from the septic shock cohort in Asan Medical Center. The primary outcome was 28-day mortality. The area under the receiver operating characteristic (ROC) curve was used to compare the predictive values of SOFA score alone and SOFA score combined with serum albumin level. **Results:** Among 5805 septic shock patients, 1529 (26.3%) died within 28 days. Mortality increased stepwise with decreasing serum albumin levels (13.6% in albumin ≥3.5, 20.7% in 3.5–3.0, 29.7% in 3.0–2.5, 44.0% in 2.5–2.0, 56.4% in <2.0). The albumin SOFA score was calculated by adding the initial SOFA score to the 4 points assigned for albumin levels. ROC analysis for predicting 28-day mortality showed that the area under the curve for the albumin SOFA score was 0.71 (95% CI 0.70–0.73), which was significantly higher than that of the initial SOFA score alone (0.68, 95% CI: 0.67–0.69). **Conclusions:** The combination of the initial SOFA score with albumin can improve prognostic accuracy for patients with septic shock, suggesting the albumin SOFA score may be used as an early mortality stratification tool.

## 1. Introduction

Sepsis is a systemic inflammatory response to infection accompanied by life-threatening organ dysfunction [1,2]. The current guideline emphasizes early recognition of organ dysfunction and early initiation of tailored therapy. However, sepsis is a complex and heterogeneous syndrome. Stratifying patients by their risk of an adverse outcome soon after admission to the emergency department (ED) or intensive care unit (ICU) can improve therapy by facilitating the development of targeted interventions and the design of clinical trials [1,2,3,4].

The sequential organ failure assessment (SOFA) score is a simple method of assessing and monitoring organ dysfunction in critically ill patients [5] and has become a crucial diagnostic tool for the new definition of sepsis, which is defined by a SOFA score ≥2 [6,7,8]. The SOFA score is traditionally calculated based on the most severe values in the 24 h preceding admission to the ICU. However, considering the updated definition of sepsis, the initial SOFA score, assessed with initial data, can be calculated.

Albumin is the primary protein involved in maintaining plasma colloid osmotic pressure and has anti-inflammatory and antioxidative characteristics. Moreover, serum albumin levels may reflect baseline host status because they can also be influenced by nutritional status and chronic disease [9,10,11]. Although hypoalbuminemia is a well-known poor outcome predictor in critically ill patients [12,13,14], further clarification is needed regarding the degree of hypoalbuminemia and its association with mortality in septic shock.

As organ dysfunction is a major determinant of sepsis, the SOFA score would be an appropriate tool for determining the severity of the disease, and its prognostic ability may be enhanced when combined with albumin. Thus, this study aimed to evaluate the prognostic ability of the initial SOFA score alone and in combination with serum albumin levels for predicting 28-day mortality in patients with septic shock at ED admission.

## 2. Materials and Methods

### 2.1. Study Design and Patients

This study was a retrospective analysis of prospectively collected data from a multicenter registry provided by the Korean Shock Society (KoSS septic shock registry) between October 2015 and May 2022 [15,16,17]. Results were validated by examining those from the septic shock cohort in Asan Medical Center (AMC) and the data from the MIMIC-IV database from the Beth Isreal Deaconess Medical Center, a tertiary academic medical center in Boston, Massachusetts, United States.

The KoSS is a collaborative research network that aims to improve the quality of sepsis diagnosis and treatment. Since October 2015, KoSS investigators have prospectively collected data from septic shock patients at the ED of 12 university-affiliated hospitals in South Korea. This registry enrolled patients aged 19 years or older with a suspected or confirmed infection and evidence of refractory hypotension or hypoperfusion [18,19]. Hypotension was defined as a systolic blood pressure (SBP) of 90 mmHg, mean arterial pressure of 70 mmHg, or a >40 mmHg decrease in SBP from baseline [20,21]. Refractory hypotension was defined as persistent hypotension despite fluid challenge (20–30 mL/kg or at least 1 L of crystalloid solution administered over 30 min). Initial resuscitation, including fluid therapy and vasopressor administration, was conducted in accordance with Surviving Sepsis Campaign guidelines. Hypoperfusion was defined as serum lactate levels ≥ 4 mmol/L. We excluded patients from the study who signed a “do not attempt resuscitation” order, did not meet the inclusion criteria within 6 h after ED arrival, were transferred from another hospital without meeting the inclusion criteria on ED arrival, or were directly transferred from EDs to other hospitals. Furthermore, patients with missing albumin levels and who were lost to follow-up were also excluded. The validation cohort, consisting of 889 patients treated between January 2016 and December 2019 in AMC, was analyzed using the same eligibility criteria as that of KoSS cohort.

A second validation cohort utilized the MIMIC-IV database, which required a data use agreement and “Protection of Human Subjects” training. We obtained access to the MIMIC-IV database on 27 October 2021. From 2008 to 2019, patients admitted to the ICU with suspicious infections were included. The presence of any of the following was considered a cause for infection suspicion: (1) A microbiology culture was performed within the first 24 h after antibiotics were prescribed on the first day of ICU admission, or (2) antibiotics were prescribed within the first 72 h after a microbiology culture on the first day of ICU admission. The primary outcome of this cohort was in-hospital mortality; therefore, we validated the model based on in-hospital mortality in this cohort. This study was approved by the institutional review boards of each participating institute, and informed consent was obtained before data collection. This study was therefore performed in accordance with the ethical standards laid down in the 1964 Declaration of Helsinki and its later amendments.

### 2.2. Data Collection

The KoSS septic shock registry case report contains standard definitions for 200 variables, including clinical characteristics, therapeutic interventions, and patient outcomes. All data were collected in a web-based electronic database by each hospital’s coordinator and anonymized through a standardized registry form. This data entry system primarily filtered out outliers or incorrect values. A quality control committee comprising emergency physicians, regional research coordinators, and investigators from all EDs was established to regularly monitor and review data quality. The committee members provided feedback to the research coordinators and investigators on the results of the quality control process, and any questions regarding data were clarified either through the use of the system’s query function or a telephone call. Demographic and clinical data, including age, sex, previous medical history, suspected infection site, initial vital signs, laboratory values on admission, severity scores, and ED interventions, were retrieved from the septic shock registry. The initial SOFA score was calculated in the ED at the time of recognition of septic shock. The primary outcome of this study was the 28-day mortality rate.

### 2.3. Statistical Analysis

Continuous variables are expressed as the mean ± standard deviation or median with the interquartile range (IQR). Categorical variables were expressed as numbers and percentages. Student’s *t*-test or the Mann–Whitney U test was used to compare continuous variables, whereas the chi-square test or Fisher’s exact test was used to compare categorical variables. We divided the albumin groups based on the percentile, observing the 10th percentile of serum albumin to be 2.1 and the 25th percentile to be 2.5. We also determined the 50th percentile to be 3.0. After using regression analysis with potential variables, we allocated the points based on the percentile of distribution and the impact size associated with the albumin level. Univariate and multivariate analyses were performed using logistic regression analysis to evaluate the association of albumin and SOFA score with 28-day mortality. Variables with *p* < 0.1 in the univariate analysis were considered in the multivariable analyses to include potential variables with clinical significance. Multivariate logistic regression analysis results were reported as odds ratios and 95% confidence intervals (CIs). Receiver operating characteristic curves (ROC) were constructed, and the area under the curve (AUC) was evaluated [22]. Using a nonparametric method, the discrimination ability of AUC between the SOFA score alone and in combination with albumin was compared. We tested the model’s accuracy using a calibration plot with risk stratification and Kaplan–Meier curves with the log-rank testing method. The performance of the model was also evaluated by the Akaike Information Criterion (AIC) and the Net Reclassification Improvement (NRI). A two-sided *p* value ≤ 0.05 was considered statistically significant. All statistical analyses were performed using SPSS for Windows version 21.0 (SPSS Inc., Chicago, IL, USA).

## 3. Results

### 3.1. Baseline Characteristics and Laboratory Findings

**Figure 1:**
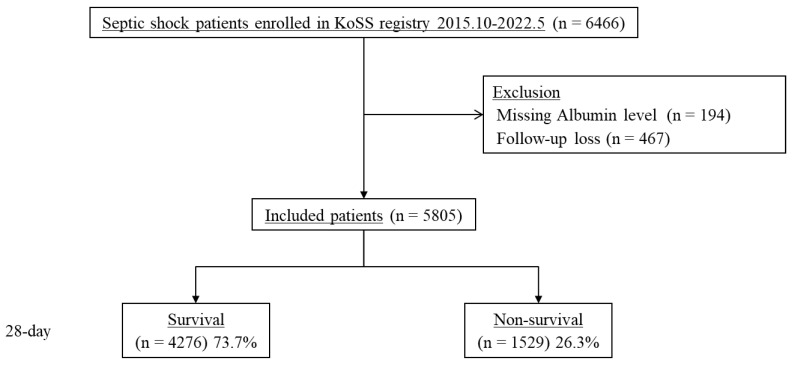
Patient flow diagram. KoSS, Korean Shock Society.

From October 2015 to May 2022, 6466 patients with septic shock were enrolled in the KoSS septic shock registry. After we excluded 194 patients with missing albumin levels and 467 patients that were lost to follow-up, we finally included 5805 patients (Figure 1). The mean age of the cohort was 68.4 years, and 3328 patients (57.3) were male. Of these, 1529 (26.3%) patients died within 28 days of admission.

Table 1 presents the baseline characteristics of the study population. The non-survivor group was older (70.7 ± 12.9 vs. 67.6 ± 13.1, *p* < 0.001) and predominantly male (60.6% vs. 56.2%, *p* = 0.003). Malignancy (33.6% vs. 24.9%, *p* < 0.001) and chronic pulmonary disease (10.3% vs. 7.4%, *p* < 0.001) were more common in the non-survivor group. Among the infection sources, pulmonary (47.6% vs. 27.7%, *p* < 0.001) and gastrointestinal (21.1% vs. 17.1%, *p* = 0.001) infections were more common in the non-survivor group, while genitourinary (29.9% vs. 20.9%, *p* < 0.001) and hepatobiliary (21.6% vs. 14.7%, *p* < 0.001) infections were more common in the survivor group. The non-survivor group had a higher heart rate and respiratory rate than the survivor group (111.5 ± 26.3 vs. 109.0 ± 25.2, *p* = 0.001; 23.8 ± 6.6 vs. 21.3 ± 5.0, *p* < 0.001; respectively). The initial SOFA score was significantly higher in the non-survivor group than in the survivor group (8.0 ± 3.4 vs. 5.9 ± 3.0, *p* < 0.001).

Table 2 shows the laboratory findings of the study population. Albumin levels were significantly lower in the non-survivor group (2.7 ± 0.6 vs. 3.1 ± 0.6, *p* < 0.001). Initial lactate level was significantly higher in the non-survivor group (6.1 ± 4.1 vs. 3.8 ± 2.8, *p* < 0.001), as was the white blood cell count and C-reactive protein.

### 3.2. Comparison of Clinical and Laboratory Characteristics in Each Group According to Albumin Levels

Table 3 represents the clinical and laboratory characteristics of the groups according to albumin levels. Within the primary cohort, the 10th percentile of serum albumin was observed to be 2.1, while the 25th percentile was found to be 2.5. Additionally, the 50th percentile was determined to be 3.0. We divided the groups according to albumin levels for every 0.5 g/dL difference. Male sex was more common as albumin levels decreased. Malignancy and liver cirrhosis were more common in the lower albumin groups, while the cardiac disease was more common in the higher albumin groups. As albumin levels decreased, hemoglobin and platelet levels also decreased significantly. However, blood urea nitrogen, creatinine, and initial lactate levels significantly increased as albumin levels increased.

**Figure 2:**
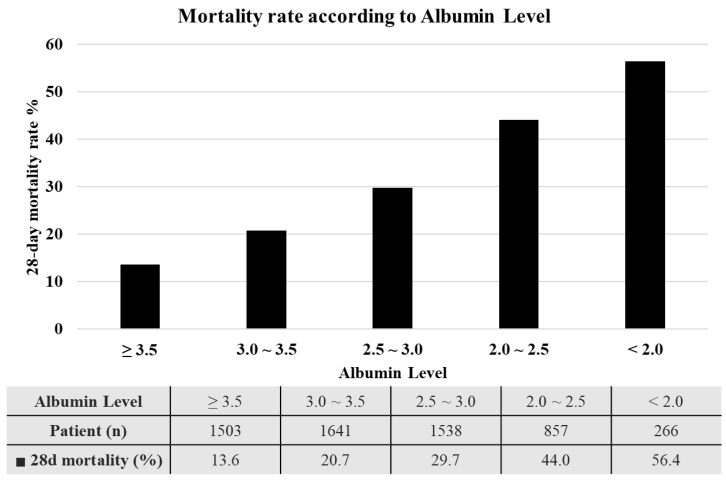
Mortality rate according to albumin levels.

The comparison of mortality rates in the groups according to albumin levels is presented in Figure 2: Group 1 (albumin ≥ 3.5 g/dL) 13.6%, Group 2 (3.5 > albumin ≥ 3.0 g/dL) 20.7%, Group 3 (3.0 > albumin ≥ 2.5 g/dL) 29.7%, Group 4 (2.5 > albumin ≥ 2.0 g/dL) 44.0%, and Group 5 (2.0 g/dL > albumin) 56.4%. In a regression analysis with other potential variables, the ORs of the defined albumin groups compared to Group 1 were 1.460 (Group 2), 2.122 (Group 3), 3.486 (Group 4), and 5.550 (Group 5) (Supplementary Table S1). Given the percentile of distribution and the impact size associated with the albumin level, we allocated 4 points according to albumin levels: 1 point for Group 2, 2 points for Group 3, 3 points for Group 4, and 4 points for Group 5.

### 3.3. Risk Factors Associated with 28-Day Mortality and Evaluation of Model Performance of the SOFA Score Combined with Albumin

Multivariate analysis was performed to identify potential risk factors for 28-day mortality, including variables with significant differences between the survivor and non-survivor groups in the univariate analysis (Table 4). SOFA score combined with albumin was independently associated with mortality (OR 1.162, 95% CI 1.135–1.190, *p* < 0.001). In the ROC curves for predicting 28-day mortality, the AUC of the SOFA score combined with albumin was 0.714 (95% CI: 0.702–0.726, *p* < 0.001), whereas that of the SOFA score alone was 0.681 (95% CI: 0.669–0.693, *p* < 0.001) and combined with lactate was 0.682 (95% CI: 0.670–694, *p* < 0.001). The predictive value of the SOFA score combined with albumin was significantly better than that of the SOFA score alone (*p* < 0.001; Supplementary Figure S1). The calibration plot with risk stratification is shown in Supplementary Figure S2. It shows that the calibration plot of SOFA score with albumin has a higher R2 of 0.991 than that of SOFA score alone of 0.889. The Kaplan–Meier curves with groups based on SOFA score alone and SOFA score combined with albumin are presented in Supplementary Figure S3. They show that the SOFA score combined with albumin might be more useful to separate the groups by survival probability.

### 3.4. Mortality Rate According to the SOFA Score Combined with Albumin

**Figure 3:**
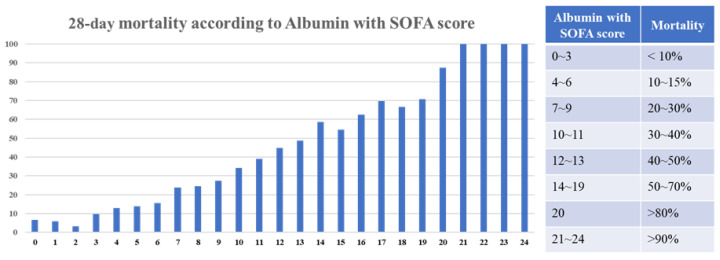
28-day mortality rate according to the SOFA score combined with albumin levels. SOFA, sequential organ failure assessment.

The distribution of the 28-day mortality rate according to the SOFA score in combination with albumin is shown in Figure 3. The mortality rate increased in proportion to the SOFA score with albumin levels. The mortality rate was approximately 20–30% when the SOFA with albumin was between 7 and 9. The 28-day mortality rate was greater than 50 percent when the SOFA score with albumin was greater than 14.

### 3.5. Validation in AMC Cohort and MIMIC-IV Database

Validation analysis included 889 septic shock patients who visited the ED at AMC between January 2016 and December 2019 (Supplementary Table S2). The mean age of the validation cohort was 65.6 years, and 521 patients (58.6) were male. The validation cohort included more patients with malignancy (47.8% vs. 27.2%) and liver cirrhosis (15.9% vs. 10.2%). Hepatobiliary infection was more common in the validation cohort, while genitourinary and pulmonary infections were less common. The 28-day and 90-day mortality rates were lower in the validation cohort. The baseline characteristics of external validation cohort 2 from the MIMIC-IV database are presented in Supplementary Table S3. The initial SOFA score was 6.6 ± 4.3 and 3943 patients (36.5%) were applied a vasopressor. The in-hospital mortality rate was 17.2%.

In Supplementary Table S4, the ability of albumin and the SOFA score to predict death at 28 days and in-hospital mortality is compared between the primary cohort and the validation cohorts. The NRI was 0.085 (0.045–0.125, *p* < 0.001), and the AIC of the SOFA score combined with albumin was lower than that of the SOFA score alone in the primary cohort. In external validation cohort 1, the AUC of the SOFA score combined with albumin was 0.714 (95% CI: 0.702–0.726, *p* < 0.001), and the SOFA score combined with albumin was independently associated with mortality (OR 1.223, 95% CI: 1.153–1.296, *p* < 0.001). For in-hospital mortality in external validation cohort 2, the AUC of the SOFA score combined with albumin was 0.787 (95% CI: 0.779–0.794, *p* < 0.001), and was independently associated with mortality.

## 4. Discussion

This study investigated whether the combination of the initial SOFA score and serum albumin levels can accurately predict 28-day mortality in septic shock patients in the early stages of the disease. Non-survivors had significantly lower albumin levels, and mortality increased inversely to albumin levels. We assigned 4 score points to patients based on their albumin level, and when combined with the initial SOFA score, it demonstrated significantly greater predictive ability than the SOFA score alone.

Despite recent advances in sepsis treatment, this condition remains a leading cause of mortality and morbidity. Early diagnosis and prompt initiation of treatment are important factors associated with improved outcomes in patients with sepsis [23]. Several biomarkers and scoring systems have been investigated to predict the clinical outcome of sepsis [24,25,26]. Further, due to the limited decision-making time in the ED, it is essential to identify patients with a high mortality risk as soon as possible to initiate antibiotic treatment and determine if they require admission to an intensive care unit, high-level care, and monitoring.

The SOFA score was created by the Working Group of the European Society of Intensive Care Medicine to describe the degree of organ dysfunction in ICU patients. Current guidelines for sepsis emphasize the importance of finding organ dysfunction, which is identified by an increase in the SOFA score from baseline [27]. In this study, the SOFA score was significantly higher in the non-survivor group, with a mean SOFA score of 8. Raith et al. demonstrated that an increase in SOFA score of 2 or more accurately predicted in-hospital mortality in ICU patients with suspected infection [7]. Furthermore, a recent meta-analysis that investigated the mortality of sepsis and septic shock observed a positive correlation between the mortality rate and the SOFA score [28]. The mortality rate for patients with a SOFA score of 8 was approximately 20–30%, according to both studies. In our study, the 28-day mortality rate was 26.3%, comparable to previous studies [29,30], and the SOFA score of the non-survivor group was also comparable.

Although organ dysfunction is a major determinant of mortality in sepsis, baseline status also plays an important role in acute illness. According to a previous study, although patients with organ dysfunction are at risk for adverse outcomes, this risk is primarily due to their poor health prior to the onset of illness [31]. Garland et al. reported that age and comorbidity contribute approximately 20% to short-term mortality compared to the characteristics of acute illness [32]. Conversely, long-term mortality was determined primarily by age and comorbidity, followed by disease severity. Furthermore, as the initial SOFA score can be influenced by the patient’s baseline status [33], it may be reasonable to predict outcomes using additional data for a more comprehensive understanding.

In this study, the non-survivor group had significantly lower albumin levels, and the mortality rate was inversely proportional to albumin levels: for every 0.5 g/dL decrease, mortality increased by approximately 10%. Albumin, a major plasma protein, has oncotic functions to maintain adequate intravascular volume. Furthermore, albumin has several beneficial properties for septic patients, including binding and transport of various endogenous molecules [34], anti-inflammatory [35] and anti-oxidative effects [36], and modulation of nitric oxidic metabolism [37]. Critical illness impacts the rate of albumin synthesis and its degradation and distribution, resulting in hypoalbuminemia [38]. Low serum albumin levels are associated with increased mortality in acute illness, including sepsis [13,39,40,41]. However, albumin levels are also associated with nutritional status, chronic disease, and disease severity. Generally, serum albumin concentration has been used as an indicator for the amount of circulating proteins in the plasma and was therefore believed to indicate nutritional status [42,43]. In sepsis, nutritional status was significantly associated with increased morbidity and mortality [44,45]. We observed that lower-albumin groups had more malignancy, chronic renal disease, and liver cirrhosis. Moreover, several laboratory findings associated with chronic illness, such as hemoglobin, blood urea nitrogen, and creatinine, which are associated with poor outcomes [46,47], differ significantly between albumin groups. Our findings were consistent with those of a previous study, which found that a low albumin level is associated with an increased risk of death.

Along with nutritional status and chronic disease, serum albumin levels are associated with frailty [48]. In critically ill patients, the presence of frailty is associated with increased risk of morbidity and mortality [49]. Furthermore, Fernando et al. found that frailty in elderly ICU patients with sepsis is linked to higher rates of hospital readmission, resource consumption, and mortality [50]. This implies that clinical frailty has a crucial role in determining the level of risk in septic patients.

To combine albumin levels and SOFA score, we assigned 4 points per 0.5 g/dL decrease in albumin level, as performed for other severe dysfunctions using the SOFA score: a 1-point increase for the group for each 0.5 g/dL reduction in albumin. We have observed the stepwise increase in the mortality according to albumin levels, so the allocated point for serum albumin would be practical to use when combined with the SOFA score. We observed a significant improvement in predictive ability compared to the SOFA score alone (0.71 [0.70–0.72] vs. 0.68 [0.67–0.69], *p* < 0.001). This is, to our knowledge, the first study to evaluate the clinical utility of the initial SOFA score in combination with albumin levels in patients with septic shock. It suggests that a more accurate prognosis prediction might be possible by combining disease severity with the factor associated with baseline status in the early stages of the disease. Furthermore, it has been demonstrated that the prevalence of hypoalbuminemia in sepsis is high and has been linked to increased mortality rates. However, there is a lack of sufficient data regarding the specific magnitude of the influence of serum albumin levels. Our research has the potential to contribute to hypothesis generation on the role of albumin in the management of sepsis.

However, caution is needed when interpreting and applying a scoring system to predict mortality. The SOFA score was initially developed to assess the degree of organ dysfunction in critically ill patients, with the advantage of reflecting disease severity in daily evaluation [5]. Thus, the score of one organ is not inherently proportional to the score of other organs due to an innate limitation of the SOFA scoring system. When the scores of each organ are incorporated, it has predictive power for mortality, as previous studies have demonstrated. Further studies are necessary to validate the prognostic usefulness of the initial SOFA score combined with albumin.

Our study has several limitations. First, as this was a multicenter study, the enrollment periods and case volumes varied by the hospital, and institutional characteristics were not adjusted for the analysis. However, all of the institutions included in this study are urban hospitals affiliated with universities. Furthermore, patients underwent protocol-driven management of septic shock. Although the distinction between patients and management characteristics would not be greater, it could be confounding factor in the heterogeneity among hospital characteristics. Second, we mainly focused on patients with septic shock in the early stages, at the ED, not in the ICU, which might have led to selection bias. Also, we defined tissue hypoperfusion as lactate levels ≥ 4 mmol/L, as our registry began before sepsis-3; if we adopted a different lactate level for tissue hypoperfusion, the study patients would be different, which could potentially impact our study’s outcome. Third, patients with a “Do Not Attempt Resuscitation” (DNR) status were excluded. As patients with DNR orders generally have more advanced and chronic diseases [51,52], which might be associated with reduced albumin levels, it could constitute a selection bias. However, there could be a possibility that the observed differences in mortality according to serum albumin were underestimated. If we included patients with DNR orders, the effect size of the combination of albumin and the initial SOFA score could be greater. Fourth, because the SOFA score was originally designed to monitor serial evaluations of organ dysfunction in ICU patients, using only the initial SOFA score to predict prognosis may be inappropriate. However, prognostic prediction is essential for establishing treatment strategies, particularly in ED, so it would be practical. Fifth, data on nutritional status, such as weight, height, and body mass index, were missing. Also, the data about clinical frailty scale were missing. These data could provide important information about the relationship between albumin and clinical frailty along with baseline nutritional status. Sixth, detailed information about previous medical history, such as cancer type, is missing. The non-survivor group has characteristics to suggest a higher prevalence of lung cancer, such as age, sex, and chronic pulmonary disease. This would be a confounding factor. Finally, data on inflammatory markers such as IL-6 and TNF were missing. The correlation between plasma albumin levels and such markers would provide an idea as to the relationship between inflammatory markers, plasma albumin, and mortality in sepsis.

## 5. Conclusions

The combination of the initial SOFA score with albumin can improve prognostic accuracy for patients with septic shock, suggesting the albumin SOFA score may be used as an early mortality stratification tool.

## Figures and Tables

**Table 1:** Baseline and clinical characteristics of the study population.

Variables	All Patients (n = 5805)	Survivors (n = 4276)	Non-Survivors (n = 1529)	*p* Value
Age, years	68.4 ± 13.1	67.6 ± 13.1	70.7 ± 12.9	<0.001
Male	3328 (57.3)	2402 (56.2)	926 (60.6)	0.003
Medical history				
Hypertension	2520 (43.4)	1859 (43.5)	661 (43.2)	0.881
Diabetes mellitus	1945 (33.5)	1431 (33.5)	514 (33.6)	0.925
Cardiac disease	867 (14.9)	627 (14.7)	240 (15.7)	0.336
Chronic pulmonary disease	475 (8.2)	318 (7.4)	157 (10.3)	0.001
Malignancy	1580 (27.2)	1066 (24.9)	514 (33.6)	<0.001
Chronic renal disease	561 (9.7)	410 (9.6)	151 (9.9)	0.762
Liver cirrhosis	590 (10.2)	414 (9.7)	176 (11.5)	0.043
Cerebrovascular disease	799 (13.8)	573 (13.4)	226 (14.8)	0.180
Source of infection				
Pulmonary	1914 (33.0)	1186 (27.7)	728 (47.6)	<0.001
Genitourinary	1600 (27.6)	1280 (29.9)	320 (20.9)	<0.001
Gastrointestinal	1055 (18.2)	733 (17.1)	322 (21.1)	0.001
Hepatobiliary	1149 (19.8)	924 (21.6)	225 (14.7)	<0.001
Unknown	367 (6.3)	263 (6.2)	104 (6.8)	0.391
Initial vital signs				
Systolic blood pressure, mmHg	99.7 ± 29.7	100.2 ± 29.7	98.4 ± 29.9	0.040
Diastolic blood pressure, mmHg	59.9 ± 18.6	60.0 ± 18.2	59.6 ± 19.6	0.483
Heart rate, per min	109.7 ± 25.5	109.0 ± 25.2	111.5 ± 26.3	0.001
Respiratory rate, per min	21.9 ± 5.7	21.3 ± 5.0	23.8 ± 6.9	<0.001
Clinical characteristics				
Initial SOFA score	6.4 ± 3.3	5.9 ± 3.0	8.0 ± 3.4	<0.001

Values are expressed as the mean ± standard deviation or number (%). SOFA, sequential organ failure assessment.

**Table 2:** Laboratory findings of the study population.

Variables	All Patients (n = 5805)	Survivors (n = 4276)	Non-Survivors (n = 1529)	*p* Value
White blood cells, ×10^3^/µL	13.0 ± 18.6	12.5 ± 13.7	14.4 ± 28.2	0.011
Hemoglobin, g/dL	10.8 ± 2.6	10.9 ± 2.5	10.4 ± 2.7	<0.001
Hematocrit, %	32.7 ± 7.7	33.0 ± 7.5	31.9 ± 8.3	<0.001
Platelets, ×10^3^/µL	164 ± 128	167 ± 126	154 ± 131	<0.001
Sodium, mmol/L	135 ± 7	135 ± 6	135 ± 8	0.012
Potassium, mmol/L	4.2 ± 0.9	4.2 ± 0.8	4.4 ± 1.0	<0.001
Chloride, mmol/L	100 ± 8	100 ± 7	100 ± 9	0.878
Blood urea nitrogen, mg/dL	35 ± 24	33 ± 22	43 ± 27	<0.001
Creatinine, mg/dL	1.9 ± 1.8	1.8 ± 1.8	2.1 ± 1.5	<0.001
Albumin, g/dL	3.0 ± 0.7	3.1 ± 0.6	2.7 ± 0.6	<0.001
AST, IU/L	136 ± 520	116 ± 485	191 ± 599	<0.001
ALT, IU/L	77 ± 269	74 ± 283	83 ± 225	0.308
Prothrombin time (INR)	1.5 ± 0.9	1.4 ± 0.8	1.6 ± 1.2	<0.001
C-reactive protein, mg/dL	16.1 ± 13.6	15.4 ± 13.0	17.9 ± 15.1	<0.001
Initial lactate, mmol/L	4.4 ± 3.3	3.8 ± 2.8	6.1 ± 4.1	<0.001
Arterial pH	7.404 ± 0.117	7.421 ± 0.098	7.359 ± 0.148	<0.001
PaCO2 (mmHg)	29.4 ± 12.2	29.0 ± 11.5	30.6 ± 14.1	<0.001
PaO2 (mmHg)	91.2 ± 45.9	89.8 ± 41.6	95.0 ± 55.7	0.001
Bicarbonate (arterial, mmol/L)	18.3 ± 6.4	18.6 ± 6.3	17.2 ± 6.6	<0.001

Values are expressed as the mean ± standard deviation or number (%). AST, aspartate transaminase; ALT, alanine transaminase; INR, international normalized ratio.

**Table 3:** Clinical and laboratory characteristics of the groups according to albumin levels.

Variables	>3.5 (n = 1503)	3.0~3.5 (n = 1641)	2.5~3.0 (n = 1538)	2.0~2.5 (n = 857)	<2.0 (n = 266)
Age, years	66.3 ± 14.5	69.5 ± 12.3	69.0 ± 12.5	69.2 ± 12.7	67.3 ± 12.2
Male	894 (59.5)	905 (55.1)	888 (57.7)	479 (55.9)	162 (60.9)
Medical history					
Hypertension	657 (43.7)	759 (46.3)	640 (41.6)	356 (41.5)	108 (40.6)
Diabetes mellitus	497 (33.1)	571 (34.8)	487 (31.7)	296 (34.5)	94 (35.3)
Cardiac disease	249 (16.6)	248 (15.1)	213 (13.8)	125 (14.6)	32 (12.0)
Chronic pulmonary disease	134 (8.9)	136 (8.3)	129 (8.4)	58 (6.8)	18 (6.8)
Malignancy	306 (20.4)	407 (24.8)	474 (30.8)	303 (35.4)	90 (33.8)
Chronic renal disease	125 (8.3)	169 (10.3)	127 (8.3)	103 (12.0)	37 (13.9)
Liver cirrhosis	95 (6.3)	135 (8.2)	188 (12.2)	124 (14.5)	48 (18.0)
Laboratory finding					
Hemoglobin, g/dL	12.2 ± 2.6	11.0 ± 2.4	10.2 ± 2.3	9.5 ± 2.3	9.1 ± 2.3
Platelet, ×10^3^/µL	180 ± 136	170 ± 123	156 ± 120	149 ± 130	128 ± 130
Blood urea nitrogen, mg/dL	30 ± 21	35 ± 22	37 ± 25	41 ± 27	41 ± 26
Creatinine, mg/dL	1.7 ± 1.5	1.9 ± 1.6	1.9 ± 2.2	1.9 ± 1.5	2.0 ± 1.5
C-reactive protein, mg/dL	12.7 ± 13.1	16.6 ± 15.0	18.1 ± 13.1	17.5 ± 12.6	15.6 ± 9.9
Initial lactate, mmol/L	4.3 ± 3.2	4.1 ± 3.2	4.4 ± 3.3	5.0 ± 3.7	5.2 ± 3.8

Values are expressed as the mean ± standard deviation or number (%).

**Table 4:** Factors associated with 28-day mortality in septic shock survivors by multivariate logistic regression analysis.

Variables	Adjusted OR	95% CI	*p* Value
Age	1.019	1.013–1.024	<0.001
Initial lactate	1.192	1.169–1.217	<0.001
Albumin with SOFA score	1.162	1.135–1.190	<0.001

Multivariate analysis included logistic regression analysis and backward elimination. OR, odds ratio; CI, confidential interval; SOFA, sequential organ failure assessment.

## Data Availability

The datasets are available from corresponding author on reasonable request.

## Supplementary Materials

The following supporting information can be downloaded at https://www.mdpi.com/article/10.3390/life14101257/s1: Figure S1: ROC curve of SOFA score combined with albumin; Figure S2: Calibration plot (240924); Figure S3: K-M graph (240924); Table S1: Adjusted odds ratios for albumin groups in regression analysis; Table S2: Comparison of characteristics of the derivation cohort and external validation cohort 1 (Asan Medical Center); Table S3: Baseline and clinical characteristics of external validation 2 cohort (MIMIC database); Table S4: Comparison of the predictive power of albumin with the SOFA score for 28-day mortality between the primary cohort and external validation cohort 1 and for in-hospital mortality in external validation cohort 2 (MIMIC data).
