# Supplementary material for: Early Mortality Stratification with Serum Albumin and the Sequential Organ Failure Assessment Score at Emergency Department Admission in Septic Shock Patients
Source: Life (Basel). 2024 Oct 2;14(10):1257. doi: 10.3390/life14101257 (PMC11509028; doi:10.3390/life14101257)
Supplement: Supplementary file 1 [file life-14-01257-s001.zip › Supplementary Fig S1. ROC curve of SOFA score combined with albumin.pdf]

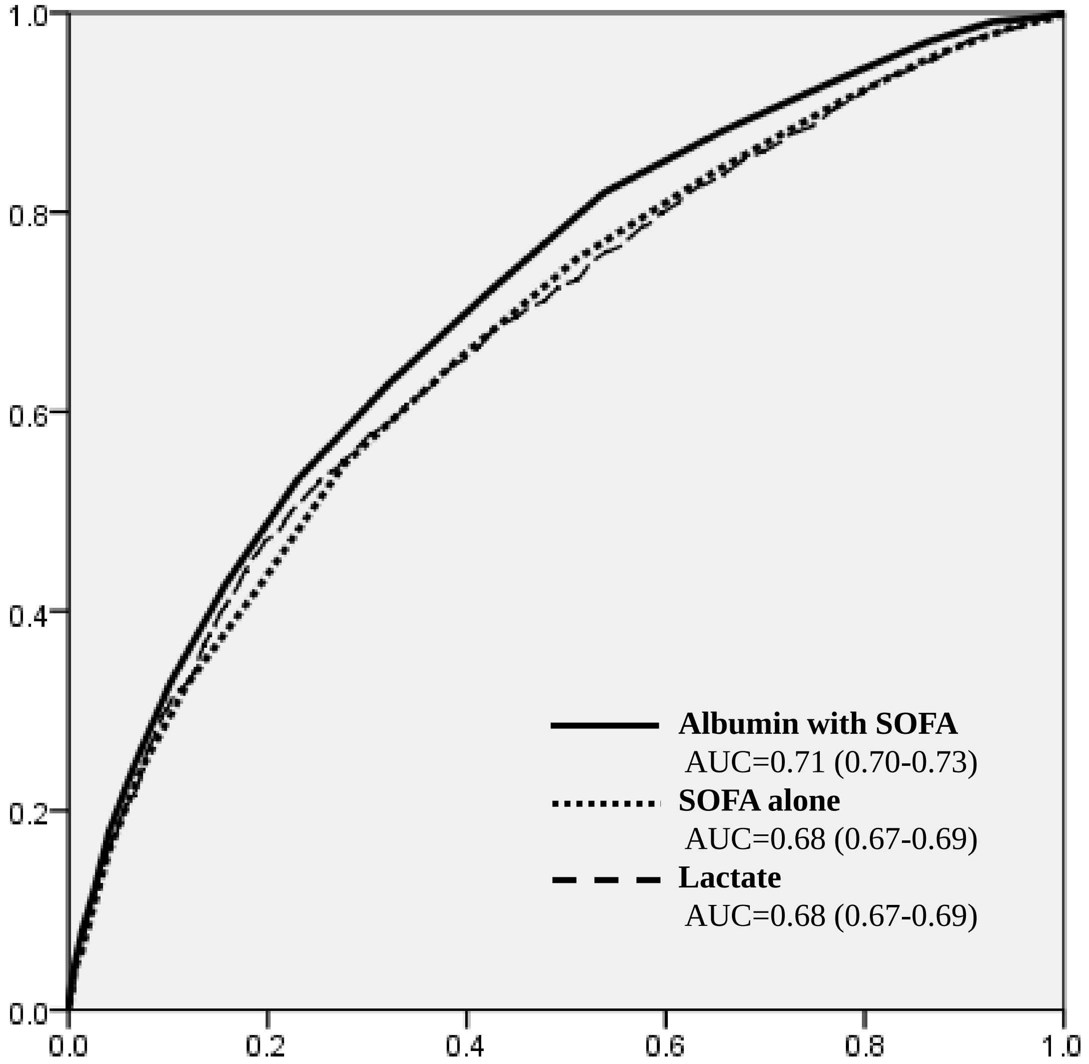

Supplementary Figure S1. The comparison of the area under the receiver operating characteristic curve between the SOFA score combined with albumin, the SOFA score alone and lactate
SOFA, sequential organ failure assessment; AUC, area under the curve
