# Supplementary material for: Early Mortality Stratification with Serum Albumin and the Sequential Organ Failure Assessment Score at Emergency Department Admission in Septic Shock Patients
Source: Life (Basel). 2024 Oct 2;14(10):1257. doi: 10.3390/life14101257 (PMC11509028; doi:10.3390/life14101257)
Supplement: Supplementary file 1 [file life-14-01257-s001.zip › Supplementary Fig S3. K-M graph (240924).pdf]

Kaplan Meier Curves according to groups based on SOFA score alone

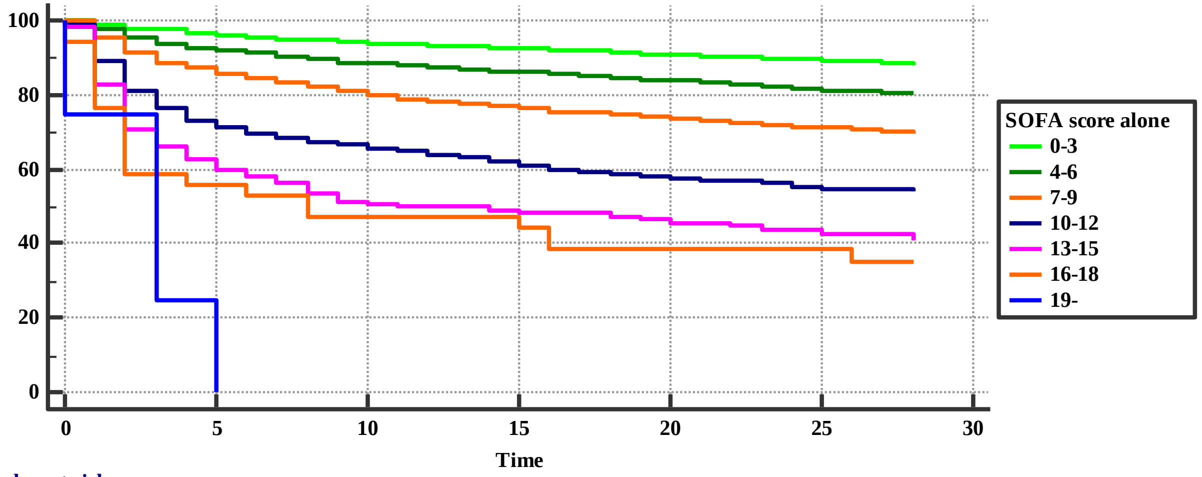

Number at risk

**Group: 0-3**	1087	1045	1021	1007	989	969	0
**Group: 4-6**	2007	1848	1775	1728	1678	1627	0
**Group: 7-9**	1613	1381	1289	1230	1183	1142	0
**Group: 10-12**	727	520	478	442	419	398	0
**Group: 13-15**	201	122	104	99	93	87	0
**Group: 16-18**	32	19	16	15	13	13	0
**Group: 19-**	3	0	0	0	0	0	0

Kaplan Meier Curves according to groups based on SOFA score combined with albumin

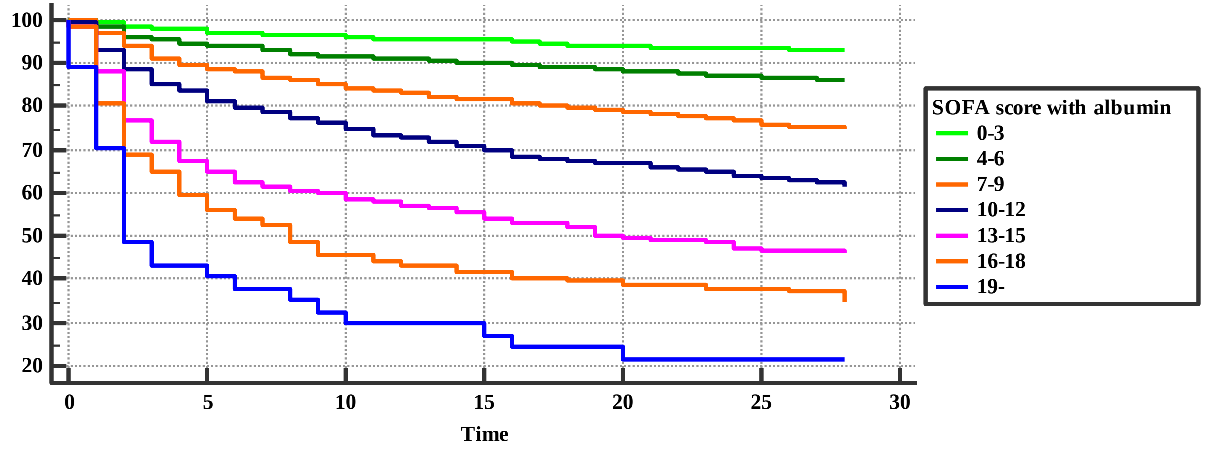

Number at risk

**Group: 0-3**	630	613	605	601	592	586	0
**Group: 4-6**	1586	1496	1453	1428	1400	1376	0
**Group: 7-9**	1718	1528	1446	1401	1357	1301	0
**Group: 10-12**	1108	905	832	773	735	698	0
**Group: 13-15**	468	306	277	254	233	218	0
**Group: 16-18**	127	72	59	54	50	49	0
**Group: 19-**	33	15	11	10	8	8	0
