# Supplementary material for: Early Mortality Stratification with Serum Albumin and the Sequential Organ Failure Assessment Score at Emergency Department Admission in Septic Shock Patients
Source: Life (Basel). 2024 Oct 2;14(10):1257. doi: 10.3390/life14101257 (PMC11509028; doi:10.3390/life14101257)
Supplement: Supplementary file 1 [file life-14-01257-s001.zip › Supplemetary Table S1.pdf]

Supplementary Table S1. Adjusted odds ratios for albumin groups in regression analysis

Variables	Adjusted OR	95% CI	*p* value
Group 1 (Albumin ≥ 3.5 g/dL)	Reference		
Group 2 (3.5> Albumin ≥ 3.0 g/dL)	1.460	1.181–1.805	<0.001
Group 3 (3.0> Albumin ≥ 2.5 g/dL)	2.122	1.724-2.611	<0.001
Group 4 (2.5> Albumin ≥ 2.0 g/dL)	3.486	2.775–4.380	<0.001
Group 5 (2.0 g/dL > Albumin)	5.550	4.010–7.680	<0.001
