# Supplementary material for: Early Mortality Stratification with Serum Albumin and the Sequential Organ Failure Assessment Score at Emergency Department Admission in Septic Shock Patients
Source: Life (Basel). 2024 Oct 2;14(10):1257. doi: 10.3390/life14101257 (PMC11509028; doi:10.3390/life14101257)
Supplement: Supplementary file 1 [file life-14-01257-s001.zip › Supplemetary Table S2.pdf]

Supplementary Table S2. Comparison of characteristics of the derivation cohort and the external validation cohort 1 (Asan Medical Center)

Characteristics	Derivation cohort (n = 5,805)	External validation 1 cohort (n = 889)
Age, years	68.4 ± 13.1	65.6 ± 12.7
Male	4,003 (57.3)	521(58.6)
**Past Medical History**		
Hypertension	2,520 (43.4)	314 (35.3)
Diabetes mellitus	1,945 (33.5)	227 (25.5)
Cardiac disease	867 (14.9)	89 (10.0)
Chronic pulmonary disease	475 (8.2)	89 (10.0)
Malignancy	1,580 (27.2)	425 (47.8)
Chronic renal disease	561 (9.7)	59 (6.6)
Liver cirrhosis	590 (10.2)	141 (15.9)
Cerebrovascular disease	799 (13.8)	48 (5.4)
**Source of infection**		
Pulmonary	1,914 (33.0)	216 (24.3)
Genitourinary	1,600 (27.6)	125 (14.1)
Gastrointestinal	1,055 (18.2)	124 (13.9)
Hepatobiliary	1,149 (19.8)	302 (34.0)
Unknown	367 (6.3)	90 (10.1)
**Initial vital signs**		
Systolic blood pressure (mmHg)	99.7 ± 29.7	92.8 ± 26.7
Diastolic blood pressure (mmHg)	59.9 ± 18.6	58.8 ± 18.0
Heart rate (per min)	109.7 ± 25.5	107.0 ± 26.1
Respiratory rate (per min)	21.9 ± 5.7	21.7 ± 4.9

**Clinical characteristics**		
Initial SOFA score	6.4 ± 3.3	5.9 ± 3.1
**Laboratory Finding**		
White blood cells, ×10³/μL	13.0 ± 18.6	11.8 ± 10.7
Hemoglobin, g/dL	10.8 ± 2.6	10.7 ± 2.4
Hematocrit, %	32.7 ± 7.7	33.4 ± 17.3
Platelets, ×10³/μL	164 ± 128	162 ± 114
Sodium, mmol/L	135 ± 7	134 ± 6
Potassium, mmol/L	4.2 ± 0.9	4.4 ± 4.3
Chloride, mmol/L	100 ± 8	98 ± 9
Blood urea nitrogen, mg/dL	35 ± 24	31.2 ± 20.3
Creatinine, mg/dL	1.9 ± 1.8	2.0 ± 2.3
Albumin, g/dL	3.0 ± 0.7	2.6 ± 0.6
AST, IU/L	136 ± 520	152 ± 517
ALT, IU/L	77 ± 269	80 ± 180
Prothrombin time (INR)	1.5 ± 0.9	1.5 ± 0.9
C-reactive protein, mg/dL	16.1 ± 13.6	14.0 ± 10.6
Initial lactate, mmol/L	4.4 ± 3.3	4.1 ± 3.1
Arterial pH	7.404 ± 0.117	7.412 ± 0.106
$PaCO_2$ (mmHg)	29.4 ± 12.2	29.3 ± 9.5
PaO_2 (mmHg)	91.2 ± 45.9	86.5 ± 40.1
Bicarbonate (arterial, mmol/L)	18.3 ± 6.4	19.4 ± 5.0
**Clinical outcomes**		
ICU admission	3,416 (58.8)	526 (59.2)
28-day mortality	1,529 (26.3)	488 (17.4)
90-day mortality	2,191 (37.7)	273 (30.7)

Values are expressed as the mean ± standard deviation or number (%).
