# Supplementary material for: Early Mortality Stratification with Serum Albumin and the Sequential Organ Failure Assessment Score at Emergency Department Admission in Septic Shock Patients
Source: Life (Basel). 2024 Oct 2;14(10):1257. doi: 10.3390/life14101257 (PMC11509028; doi:10.3390/life14101257)
Supplement: Supplementary file 1 [file life-14-01257-s001.zip › Supplemetary Table S3.pdf]

Supplementary Table S3. Baseline and clinical characteristics of the external validation 2 cohort (MIMIC database)

Characteristics	External validation 2 (MIMIC database) (n = 10,796)
Age	64.6 ± 16.5
Male	6,140 (56.9)
Charlson Comorbidity Index	5.7 ± 3.0
White blood cells, $\times 10^3/\mu L$	13.3 ± 12.2
Hemoglobin, g/dL	11.4 ± 2.4
Platelets, $\times 10^3/\mu L$	189 ± 92
Creatinine, mg/dL	1.6 ± 1.6
Bilirubin, mg/dL	2.4 ± 5.3
Albumin, g/dL	3.2 ± 0.7
Initial lactate, mmol/L	3.2 ± 3.1
First day ventilator	5,386 (49.9)
Renal replacement therapy	365 (3.4)
Vasopressor	3,943 (36.5)
Initial SOFA	6.6 ± 4.3
In-hospital mortality	1,860 (17.2)
