# Supplementary material for: Early Mortality Stratification with Serum Albumin and the Sequential Organ Failure Assessment Score at Emergency Department Admission in Septic Shock Patients
Source: Life (Basel). 2024 Oct 2;14(10):1257. doi: 10.3390/life14101257 (PMC11509028; doi:10.3390/life14101257)
Supplement: Supplementary file 1 [file life-14-01257-s001.zip › Supplemetary Table S4.pdf]

Supplementary Table S4. Comparison of the predictive power of albumin with the SOFA score for 28-day mortality between the primary cohort and the external validation cohort 1 and for in-hospital mortality in external validation cohort 2 (MIMIC data)

External validation 1 for 28-day mortality

Characteristics		Primary cohort	p-value	External validation 1	p-value
AUC	SOFA with albumin	0.714 (0.702–0.726)	<0.001	0.722 (0.691–0.751)	<0.001
	SOFA alone	0.682 (0.670–0.694)		0.683 (0.652–0.714)	
NRI	SOFA with albumin	0.085 (0.045-0.125)	<0.001	0.038 (-0.084-0.160)	0.542
	SOFA alone				
AIC	SOFA with albumin	10266.528		-1266.88	
	SOFA alone	10668.108		-1265.18	
Adjusted OR [95% CI]		1.162 (1.135–1.190)	<0.001	1.223 (1.153–1.296)	<0.001

External validation 2 for in-hospital mortality

Characteristics		Primary cohort	p-value	External validation 2	p-value
AUC	SOFA with albumin	0.713 (0.701–0.724)	<0.001	0.787 (0.779–0.794)	<0.001
	SOFA alone	0.678 (0.665–0.690)		0.775 (0.767–0.783)	
Adjusted OR [95% CI]		1.652 (1.557–1.754)	<0.001	1.167 (1.132–1.203)	
