# Supplementary figures and images for: Early Mortality Stratification with Serum Albumin and the Sequential Organ Failure Assessment Score at Emergency Department Admission in Septic Shock Patients
Source: Life (Basel). 2024 Oct 2;14(10):1257. doi: 10.3390/life14101257 (PMC11509028; doi:10.3390/life14101257)

**Calibration plot of SOFA score alone**

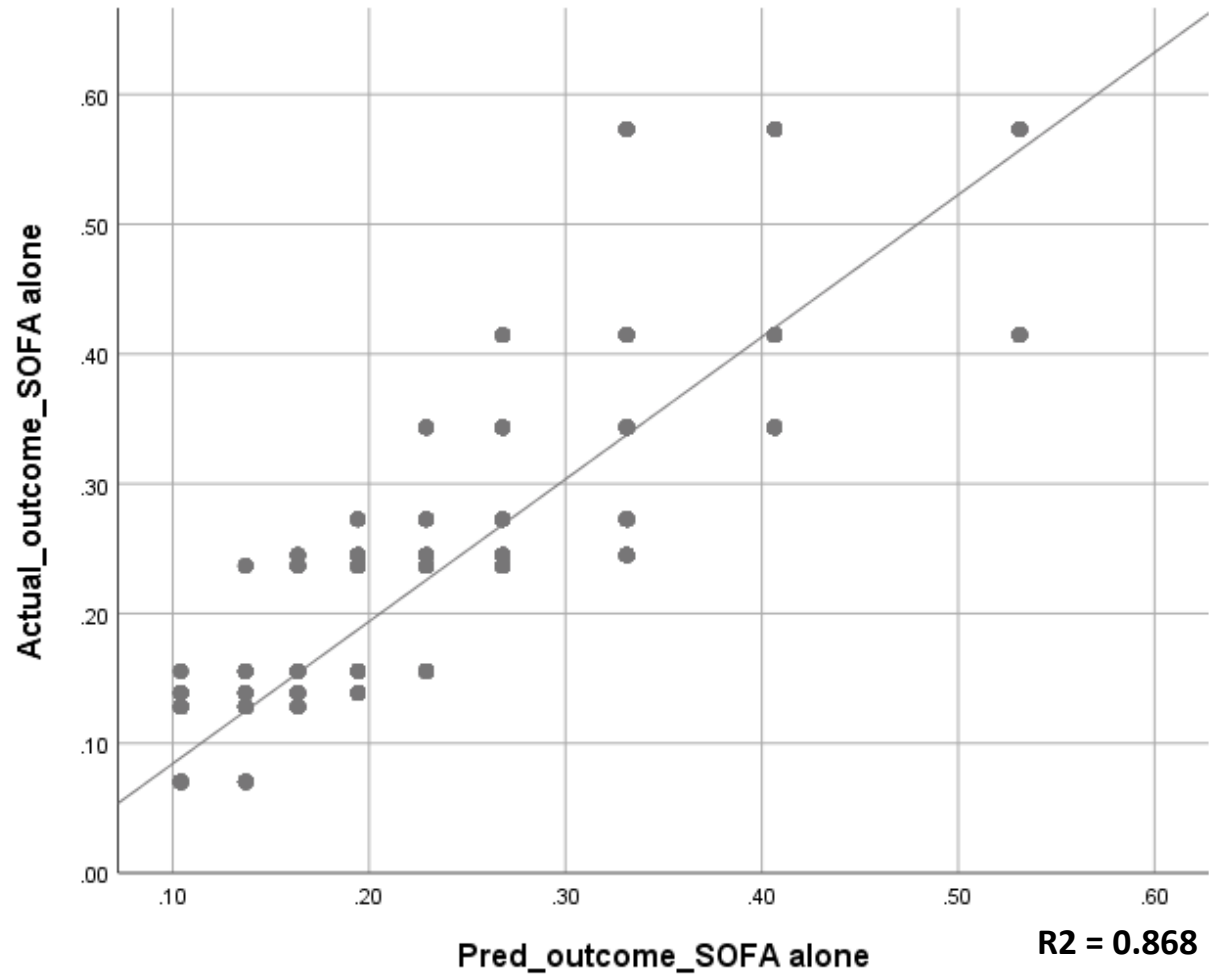

**Calibration plot of SOFA score combined with albumin**

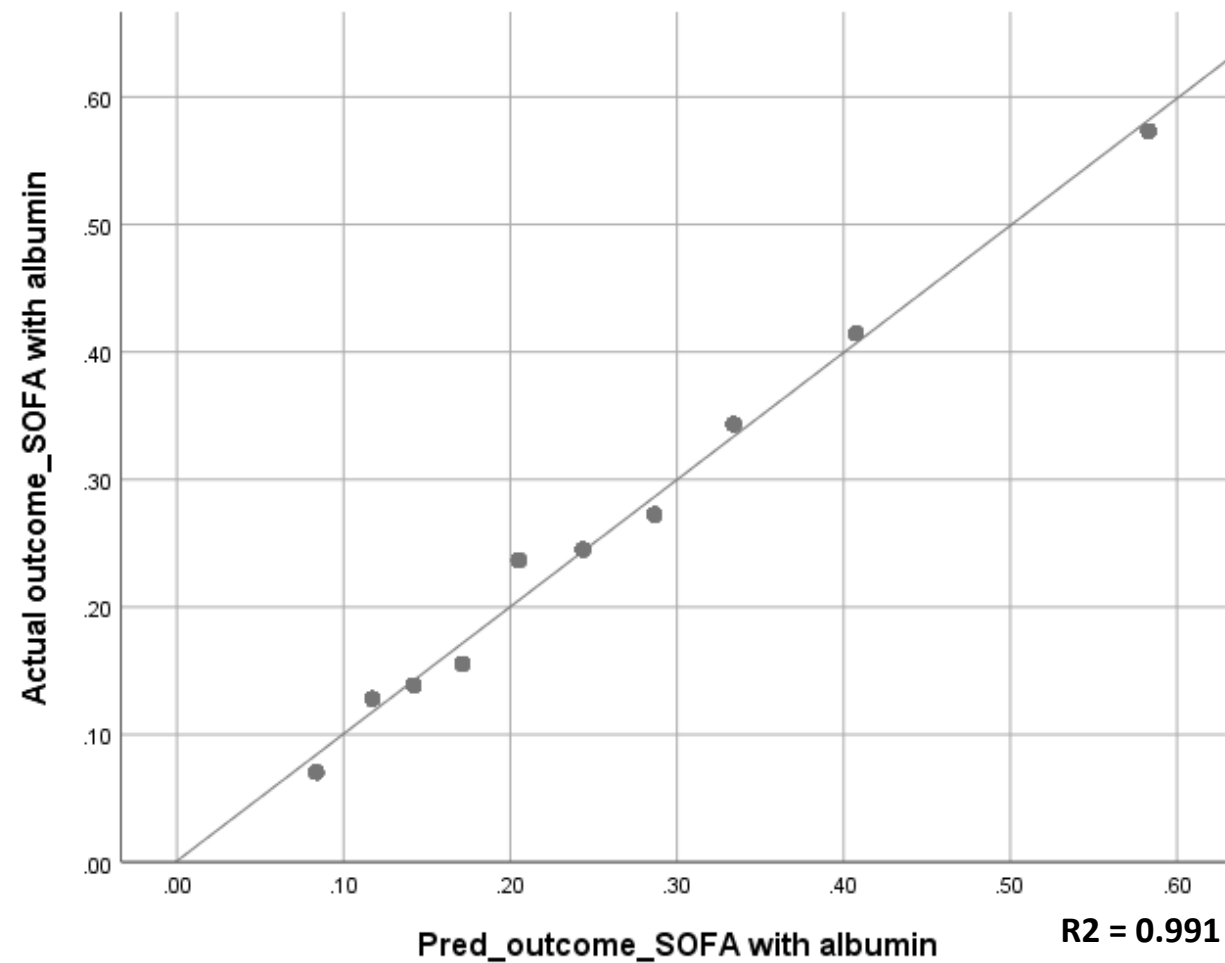

Supplement: Supplementary file 1 [file life-14-01257-s001.zip › Supplementary Fig S2. Calibration plot (240924).pdf]
